# Nitazoxanide Analogs: Synthesis, In Vitro Giardicidal Activity, and Effects on *Giardia lamblia* Metabolic Gene Expression

**DOI:** 10.3390/ijms26104504

**Published:** 2025-05-08

**Authors:** Laura Morales-Luna, Beatriz Hernández-Ochoa, Abigail González-Valdez, Montserrat Vázquez-Bautista, Roberto Arreguin-Espinosa, Verónica Pérez de la Cruz, Sergio Enríquez-Flores, Ignacio De la Mora De la Mora, Elizabeth Hernández-Urzúa, Rosa Angélica Castillo-Rodríguez, Noemí Cárdenas-Rodríguez, Víctor Martínez-Rosas, Gabriel Navarrete-Vázquez, Saúl Gómez-Manzo

**Affiliations:** 1 Laboratorio de Bioquímica Genética, Instituto Nacional de Pediatría, Secretaría de Salud, Mexico City 04530, Mexico; lauraeloisamorales@ciencias.unam.mx (L.M.-L.); montsevazquez97@gmail.com (M.V.-B.); 2 Posgrado en Ciencias Biológicas, Universidad Nacional Autónoma de México, Mexico City 04510, Mexico; 3 Laboratorio de Inmunoquímica, Hospital Infantil de México Federico Gómez, Secretaría de Salud, Mexico City 06720, Mexico; beatrizhb_16@comunidad.unam.mx; 4 Departamento de Biología Molecular y Biotecnología, Instituto de Investigaciones Biomédicas, Universidad Nacional Autónoma de México, Mexico City 04510, Mexico; abigaila@biomedicas.unam.mx; 5 Programa de Posgrado en Biomedicina y Biotecnología Molecular, Escuela Nacional de Ciencias Biológicas, Instituto Politécnico Nacional, Mexico City 11340, Mexico; 6 Departamento de Química de Biomacromoléculas, Instituto de Química, Universidad Nacional Autónoma de México, Mexico City 04510, Mexico; arrespin@unam.mx; 7 Neurobiochemistry and Behavior Laboratory, National Institute of Neurology and Neurosurgery “Manuel Velasco Suárez”, Mexico City 14269, Mexico; veped@yahoo.com.mx; 8 Laboratorio de Biomoléculas y Salud Infantil, Instituto Nacional de Pediatría, Secretaría de Salud, Mexico City 04530, Mexico; sergioenriquez@ciencias.unam.mx (S.E.-F.); ignaciodelamora@ciencias.unam.mx (I.D.l.M.D.l.M.); 9 Laboratorio de Toxicología Genética, Instituto Nacional de Pediatría, Secretaría de Salud, Mexico City 04530, Mexico; elyzabet91@yahoo.com.mx; 10 Centro de Investigación en Ciencia Aplicada y Tecnología Avanzada (CICATA) Unidad Morelos, Instituto Politécnico Nacional, Boulevard de la Tecnología, 1036 Z-1, P 2/2, Atlacholoaya 62790, Mexico; racastillo@ipn.mx; 11 Laboratorio de Neurociencias, Instituto Nacional de Pediatría, Secretaría de Salud, Mexico City 04530, Mexico; noemicr2001@yahoo.com.mx; 12 Departamento de Ingeniería Química y Bioquímica, Instituto Tecnológico de Milpa Alta, Tecnológico Nacional de México, Milpa Alta, Mexico City 12300, Mexico; ing_vicmr@hotmail.com; 13 Facultad de Farmacia, Universidad Autónoma del Estado de Morelos, Av. Universidad 1001, Chamilpa, Cuernavaca 62209, Mexico

**Keywords:** nitrothiazole, antigiardial activity, *Giardia lamblia*, gene expression

## Abstract

Giardiasis is a common intestinal infection caused by *Giardia lamblia*. The standard treatment for this parasitic infection involves the administration of nitroimidazoles, albendazoles, and nitrothiazoles. However, in recent years, *Giardia lamblia* strains resistant to these treatments have been reported. Additionally, the current therapies exhibit considerable side effects, highlighting the need for new compounds that specifically target this parasite. The aim of this study was to evaluate nitrothiazole analogs and assess their impact on the metabolic, redox, and structural gene expression of this parasite. First, the compounds CNZ-7, CNZ-8, FLP-2, FLP-6, and FLP-8 were tested at concentrations ranging from 0 to 50 µM to determine their IC_50_ in *G. lamblia* cultures. Subsequently, gene expression changes and structural cell damage in trophozoites were analyzed following incubation with the IC_50_ of each compound. The giardicidal activity of the compounds was also evaluated in a nitazoxanide-resistant strain. The results showed that FLP-2, FLP-6, and FLP-8 exhibited a stronger effect on trophozoite viability compared to nitazoxanide (NTZ) and metronidazole (MTZ). Both compounds induced an increase in the expression of phosphofructokinase (*PFK)*, glyceraldehyde-3-phosphate dehydrogenase *(GAPDH)*, pyruvate kinase *(PK)*, pyruvate phosphate dikinase *(PPDK)*, and pyruvate:ferredoxin oxidoreductase (*PFOR)*. Additionally, FLP-2 caused ultrastructural alterations in trophozoites. Furthermore, FLP-2, FLP-6, and FLP-8 demonstrated efficacy against drug-resistant strains. These findings suggest that FLP-2, FLP-6, and FLP-8 are promising candidates for the treatment of giardiasis, as they effectively reduce parasite viability, modify gene expression, and exhibit activity against drug-resistant *G. lamblia* strains.

## 1. Introduction

Giardiasis is an intestinal disease caused by the parasite *Giardia lamblia*, generally related to traveler’s disease [1]. It has high morbidity rates of 30 to 60% in the population living in developing areas [2,3], where the most affected populations are people with compromised immune systems, preschool-aged children, and people living in rural areas, and the last two are associated with poor hygiene habits in addition to the absence of basic sanitary measures [2,4]. Giardiasis has clinical relevance in infants since their immune system is not sufficiently capable of responding to this infection. Moreover, in patients under 5 years of age with a compromised immune system due to diseases such as HIV or cancer, giardiasis triggers serious clinical manifestations such as persistent diarrhea, intestinal malabsorption, and marked weight loss that compromise physical and cognitive development [2,5].

The World Health Organization (WHO) included giardiasis in the initiative of neglected and water quality-associated diseases [6,7,8]. Due to the high incidence of the disease, *Giardia lamblia* has been classified as the third most common biological agent causing diarrheal diseases, with more than 280 million clinical cases diagnosed annually, surpassed only by rotavirus and *Cryptosporidium parvum* and *Cryptosporidium hominis* [9].

The pharmacological treatment of giardiasis consists of the administration of nitroimidazoles such as metronidazole, tinidazole, secnidazole, and ornidazole. These drugs, in their reduced forms, modify the helical structure of the parasite’s DNA by breaking its strands and causing a loss of its functions, resulting in cellular apoptosis [10,11,12,13]. In addition, albendazole is often used as an alternative, whose mechanism of action entails the depolymerization of the β-tubulin microtubules. Nitrothiazoles, such as nitazoxanide, are also used, showing 71% efficacy; their mechanisms of action involve the non-competitive inhibition of the enzymes pyruvate:ferredoxin oxidoreductase (PFOR) and glucose-6-phosphate dehydrogenase::6-Phosphogluconolactonase (G6PD::6PGL) [11,12,13,14].

Various adverse effects have been reported for the prescribed treatments of giardiasis, including metronidazole-induced encephalopathy, and other adverse effects such as headaches, a loss of reflexes, a lack of coordination in the limbs when walking, and a loss of coordination when speaking, among others, have been described [15]. The increase in the number of *Giardia lamblia* strains resistant to antigiardial drugs has been widely documented. This resistance is related to mutations in key metabolic pathways of the parasite and its ability to employ evasion mechanisms, such as the formation of cysts or the modification of essential proteins in the target pathways [13,16,17,18]. This phenomenon underlines the urgent need to develop new therapeutic alternatives.

Therefore, the search for new drugs is of utmost importance. Identifying new molecules or therapeutic combinations that the parasite is not resistant to is a priority of biomedical research. The study of the specific metabolic pathways of *G. lamblia* and its survival and virulence mechanisms offers opportunities for the development of targeted treatments. In addition, modern tools, such as bioactive compound screening, structural biology, and genetic editing, are driving the identification of new therapeutic targets [13].

The study of anaerobic metabolism in a-mitochondrial parasites such as *G. lamblia* has led to the discovery of potential therapeutic targets for developing new drugs. In this regard, compounds that disrupt glucose catalysis pathways could reduce the viability of *G. lamblia* [19,20]. Glucose is metabolized in glycolytic and pentose phosphate pathways (PPP). The PPP has an oxidative phase, where NADPH molecules are generated. The PPP has been described as a relevant pathway of this parasite since the NADPH molecules produced in the oxidative phase are used to respond to the oxidative stress produced as a host response to the parasitic infection [21], also known as the NADPH. This plays an essential role in the synthesis of fatty acids and in maintaining redox homeostasis, as it facilitates the elimination of reactive oxygen species through the thioredoxin/peroxiredoxin systems [22,23,24]. In *G. lamblia*, glucose-6-phosphate dehydrogenase: 6-phosphogluconolactonase (G6PD: 6PGL) is the first enzyme of the PPP, responsible for catalyzing the first two reactions of the pathway; it has bifunctional activity and since G6PD: 6PGL provides substrates for nucleotide synthesis and NADPH as a source of reducing equivalents, it has been considered an antiparasitic pharmacological target [25,26,27]. A comparative analysis of the fused G6PD::6PGL enzyme of G. lamblia with the crystallographic structure of human G6PD (HsG6PD) showed a sequence identity of 35% [27].

In this work, we report newly synthesized compounds whose therapeutic target is the fused enzyme of *Giardia lamblia* and demonstrate their selective effect on *G. lamblia* trophozoite. Our work offers new approaches for the treatment of giardiasis; furthermore, due to the epidemiological impact of giardiasis, the progress in the development of innovative treatments will be crucial to mitigate its impact on global health.

## 2. Results and Discussion

### 2.1. Evaluation of the Concentration–Response Effect of the Compounds on the Viability of Giardia lamblia

**Figure 1:**
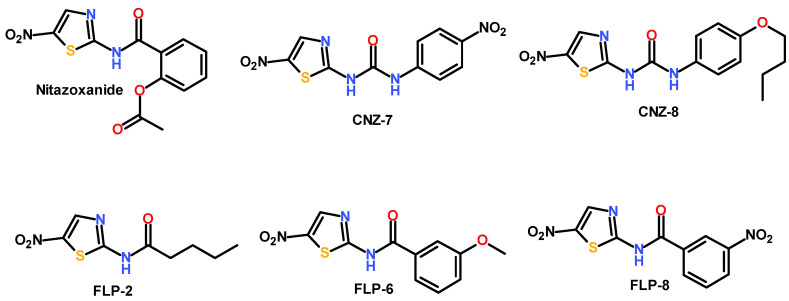
Chemical structures of compounds CNZ-7, CNZ-8, and FLP-2, the inhibitors of the G6PD enzyme, and FLP-6 and FLP-8, the inhibitors of the viability of *G. lamblia* cultures. All five compounds are analogs of nitazoxanide. The structural region shared by the compounds comprises the thiazole ring linked to a nitro group followed by the urea or amide substituents.

Previously, our working group identified three antigiardial compounds whose therapeutic target is the G6PD::6PGL enzyme of the *G. lamblia* parasite [28]; the compounds are named CNZ-7, CNZ-8, and FLP-2 and are analogs of the drug nitazoxanide. Their structure has a nitrothiazole ring; CNZ-7 and CNZ-8 have a urea group, while FLP-2 has a pentanamide substituent (Figure 1). In addition, we selected two compounds, FLP-6 and FLP-8, which are not inhibitors of the enzyme, but at a concentration of 25 µM, they decreased the viability of the parasite by 100%. The five compounds share the nitrothiazole structure composed of the thiazole ring and the -NO_2_ group attached to the 5-position of the ring, and this structure is relevant since the antimicrobial and antiparasitic activity of these compounds is attributed to it [29]. In contrast, an amide group is attached at the 2-position, which is the group that links the nitrothiazole ring with the rest of the groups of the molecules.

**Figure 2:**
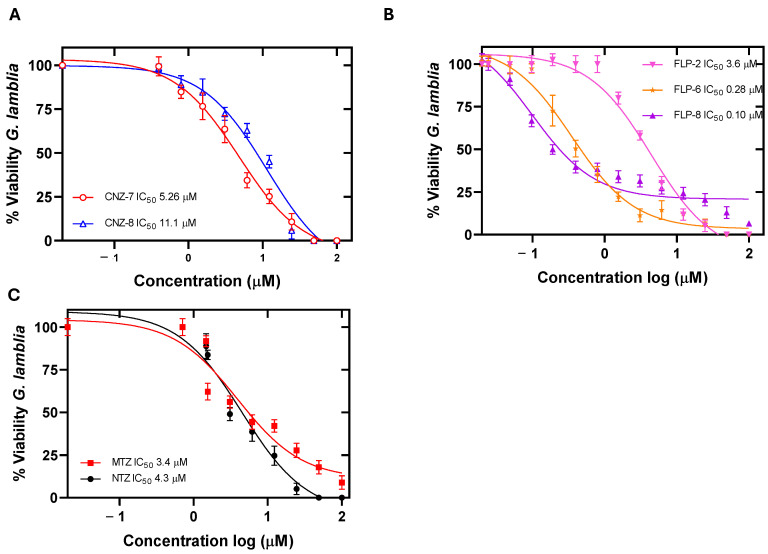
Growth curves of *Giardia lamblia* culture in the presence of (**A**) compounds CNZ-7 and CNZ-8. (**B**) compounds FLP-2, FLP-6, and FLP-8 and (**C**) MTZ and NTZ. Trophozoites were incubated in the presence of compounds for 48 h at 37 °C with increasing concentrations of each compound, and the IC_50_ concentration (µM) of each compound was subsequently determined using the IC_50_ Calculator tool online (https://www.aatbio.com/tools/ic50-calculator) (accessed on 24 November 2024). Values represent the mean ± standard deviation of three independent experiments.

The effect of different concentrations of the compounds on the viability of *G. lamblia* trophozoite culture was evaluated after 48 h of incubation at 37 °C. We determined the IC_50_ value, and we observed a concentration-dependent relationship, where the viability of the trophozoites decreased at higher concentrations of the compounds (Figure 2) compared to the drugs nitazoxanide and metronidazole. The results showed that the compounds CNZ-7 and CNZ-8 showed lower potency, with IC_50_ values of 5.2 µM and 11.1 µM, respectively (Figure 2A). While the compounds FLP-6 and FLP-8 presented the most effective outcome as their IC_50_ values were 0.28 µM and 0.1 µM, respectively, followed by FLP-2 with an IC_50_ value of 3.6 µM (Figure 2B), which is a similar value to the IC_50_ calculated for the drugs NTZ and MTZ (3.4 µM and 4.3 µM, respectively) (Figure 2C). FLP-6 presented a 12 times greater relative potency than NTZ and 15 times greater than MTZ; in contrast, FLP-8 presented 34 and 43 times greater potency than NTZ and MTZ, respectively. Therefore, these compounds could be used as an alternative for the treatment of patients with giardiasis caused by a resistant strain.

The calculated IC_50_ values of the studied molecules are lower than those of other previously reported molecules that inhibit the viability of *G. lamblia* cultures [19,30,31]; thus, we can interpret that the studied compounds have a more significant effect at lower concentrations, even at nanogram concentrations, as is the case of FLP-6 and FLP-8, which are promising antigiardial molecules.

### 2.2. Cytotoxicity and ADMET Predictive Parameters of the Compounds

The cytotoxic effects and the absorption, distribution, metabolism, excretion, and toxicity (ADMET) predictive parameters of the compounds CNZ-7, CNZ-8, and FLP-2 have been previously determined [28]; however, those of compounds FLP-6 and FLP-8 have not yet been evaluated. Thus, the ADMET parameters of the compounds were evaluated using bioinformatics programs and compared with the parameters of the drug of choice, nitazoxanide. Furthermore, the cytotoxicity of FLP-6 and FLP-8 in human cancer cell lines, Caco-2 (ATCC-HTB-37) and HT-29 (ATCC-HTB-38), both of colorectal carcinoma, was determined.

In the development of new drugs, the prediction of ADMET parameters is relevant since they predict the safety and efficacy of drugs when administered orally. Table 1 shows the ADMET values determined for the five compounds and for NTZ. It is observed that all the compounds meet the acceptable criteria for pharmacokinetic parameters. Absorption values were acceptable for gastrointestinal absorption and permeability of Caco-2 cells, which have been widely used as a model for drug permeability. The binding to plasma protein values was greater than 90% for all compounds, including NTZ; therefore, it can be predicted that less than 10% of the compounds remain available for efficacy, which is similar to that calculated for NTZ. Regarding the permeability of the blood–brain barrier, FLP-2 and NTZ do not permeate the blood–brain barrier. Regarding the volume of distribution, good values are predicted for the five compounds. The compounds were found to be substrates of cytochromes CYP3A4 and CYP2D, both of which are relevant in drug metabolism, since the compounds depend on these enzymes for their metabolism. Enzyme activity and genetic variants may affect their efficacy and safety.

Clearance and half-life are two pharmacokinetic parameters related to how a drug is eliminated from the body and the time it remains in the body. These parameters are essential to define the frequency and dose of administration of a drug to reach and maintain adequate therapeutic concentrations [32]. The compounds showed acceptable values of clearance; thus, it is possible to infer that the compounds were excreted, resulting in a short half-life. Finally, the toxicity parameters showed that the compounds presented a very low blockage of the hERG channel and could be considered non-cardiotoxic molecules. They exhibited moderate oral toxicity in rats. Regarding carcinogens, although some compounds showed positive values, it is important to highlight that these bioinformatic studies, in general, only allow us to predict the possible behavior of the compounds. It is important to experimentally verify their possible effects in animal models to determine these parameters and thus ensure that the compounds are safe for therapeutic use [32].

**Figure 3:**
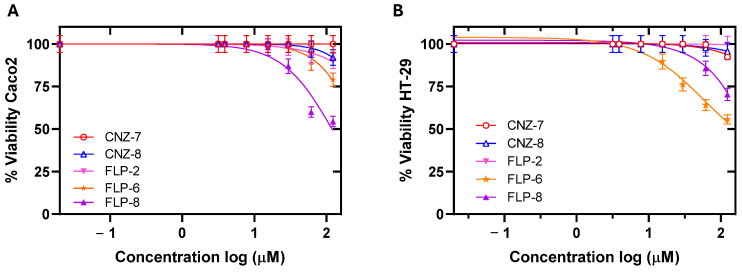
Cytotoxic effect of compounds on Caco-2 and HT-29 cells. The concentration–response curves for cell viability of (**A**) Caco-2 cells and (**B**) HT-29 cells treated with CNZ-7, CNZ-8, FLP-2, FLP-6, and FLP-8. Cell proliferation analysis was performed using an XTT viability assay. Values represent mean ± SD of three independent experiments, with standard errors of less than 5%.

Cytotoxicity was evaluated in two cancer cell lines belonging to human intestinal cell strains to determine the possible adverse effects of the five compounds in humans. It was determined that the compounds at concentrations of less than 10 µM did not cause a decrease in the viability of both cell lines (Figure 3); however, at concentrations of 125 µM, the compounds CNZ-7, CNZ-8, and FLP-2 decreased cell viability by up to 20%, and FLP-6 and FLP-8 decreased cell viability by up to 30 and 50%, respectively. The median concentration cytotoxic value (CC_50_), a parameter used in cytotoxicity studies to know the concentration of a compound that causes the death of 50% of the cells in a culture at a specific time, was determined [32]. The compounds showed CC_50_ values higher than the IC_50_ values calculated in *G. lamblia* trophozoites; the CC_50_ value of FLP-6 and FLP-8 for the HT-29 cell line was 115.3 µM and 43.6 µM, respectively, while the CC_50_ value of FLP-6 and FLP-8 for Caco-2 cells was 178.1 µM and 46 µM, respectively (Table 2).

In addition, the IC_50_ and CC_50_ values were used to calculate the selectivity index, which indicates how selective the compounds are for binding to one type of cell over another. The five compounds exhibited high selectivity values for Giardia trophozoites (Table 2), suggesting that the compounds are effective against their target cell without being toxic to human cells.

Based on the presented data, it can be established that compounds CNZ-7, CNZ-8, FLP-2, FLP-6, and FLP-8 are very potent compounds with IC_50_ values in the low micromolar range, i.e., less than 15 µM, and some compounds even have values less than 1 µM, and they also have a high selectivity index. In this sense, there are few compounds reported so far that have presented low IC_50_ values in culture and have low toxicity. Furthermore, the cytotoxicity studies of giardicidal compounds have been reported in a variety of human cell lines such as fibroblasts, blood cells, and even non-human cells such as VERO cells derived from the kidney of an African green monkey, a species phylogenetically close to humans [29,31,33,34]. In this study, cytotoxicity was evaluated in Caco-2 and HT-29 cell lines as models of small intestinal microenvironments due to their ability to adopt enterocyte morphology and to form a monolayer like a layer of the small intestine. The Caco-2 and HT-29 cells have also been used for studying the parasite–enterocyte interaction in the intestine of patients during an infection [35,36].

**Table 2:** The in vitro giardicidal activity and selective cytotoxicity of compounds. The numbers in parentheses correspond to the ratio of CC_50_/IC_50_ values between mammalian cells and trophozoite ATCC strain 30957 (selectivity index).

Compound	IC_50_ (µM)		CC_50_ (µM)	
	*G. lamblia*	*G. lamblia* (NTZ Resistant)	HT-29 (SI)	Caco-2 (SI)
CNZ-7	5.26	2.5	529 (100)	640 (121)
CNZ-8	11.1	13.7	622 (56)	633 (57)
FLP-2	3.6	0.37	1912 (531)	3184 (884)
FLP-6	0.28	0.54	115.33 (411)	178.18(636)
FLP-8	0.10	1.19	43.67 (436)	46 (460)
MTZ	3.4	Not determined	550 (161) [37]	545 (160) [37]
NTZ	4.3	7.29	634 (147) [37]	580 (134) [37]

Notably, FLP-2 has an IC_50_ similar to MTZ and 1.2 times lower than NTZ, while FLP-6 and FLP-8 have an IC_50_ lower than MTZ and NTZ, the drugs used in giardiasis treatment, where the potency of FLP-6 is 12 and 15 times greater than that of MTZ and NTZ, respectively, and FLP-8 has a potency of 34 and 43 times greater than that of MTZ and NTZ, respectively. Therefore, future studies on these compounds as potential therapeutic agents may offer alternatives to the currently available antigiardial drugs.

### 2.3. Effect on Metabolic Gene Expression Levels of Giardia lamblia

Glycolysis is key in the energy metabolism of *G. lamblia*, partly due to the absence of mitochondria in this parasite. In addition, *G. lamblia* has mechanisms that increase the efficiency of glycolysis, such as those involving the enzymes pyruvate phosphate dikinase (*PPDK*) and an inorganic phosphate-dependent phosphofructokinase (*PFK*). These factors underline the importance of glycolysis for the parasite [38,39,40]. We evaluated the expression of genes encoding glycolysis proteins in order to elucidate whether the effects caused by the compounds on parasite viability are related to changes in the expression of these genes. The gene expression assay was performed using RT-qPCR to evaluate the transcription levels of several genes in trophozoites that were incubated for 48 h at the IC_50_ concentration for each compound.

**Figure 4:**
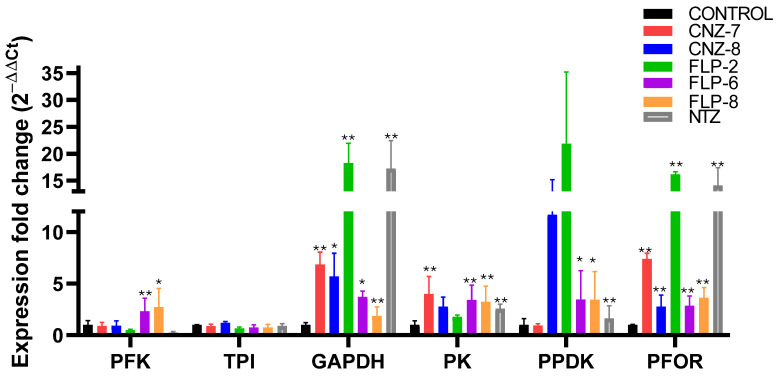
Relative expression of glycolytic genes in *Giardia lamblia* determined using RT-qPCR. Gene expression was compared between parasites cultured without treatment (negative control) and trophozoites exposed to compounds, using Aldolase as a reference gene. Each color corresponds to the evaluation of different compounds: red—CNZ-7; blue—CNZ-8; green—FLP-2; purple—FLP-6; orange—FLP-8; gray—NTZ; and black—negative control. The asterisk (*) indicates a significant difference (*p* < 0.05) in expression, and double asterisk (**) indicates a significant difference (*p* < 0.001) in expression. Values represent the mean ± SD of three replicates.

The results indicate a differential expression of several genes related to metabolism after incubation with the five compounds. As seen in Figure 4, the gene expression of *PFK* showed a reduction in trophozoites when exposed to CNZ-7, CNZ-8, FLP2, and NTZ, although the decrease was not statistically significant compared to the control group, while FLP-6 and FLP8 induced a significant overexpression with 2.3- and 2.7-fold changes in the expression of the *PFK* gene. With respect to the expression of the triosephosphate isomerase *(TPI)* gene, the compounds and NTZ drug do not induce a significant change. In contrast, trophozoites treated with all compounds and NTZ showed increased levels of glyceraldehyde-3-phosphate *(GAPDH)*, pyruvate kinase (*PK)*, pyruvate phosphate dikinase (*PPDK*), and pyruvate:ferredoxin oxidoreductase (*PFOR*) gene expression. The *GAPDH* gene encoding a regulatory enzyme of the glycolysis pathway showed significant overexpression (*p* < 0.001) with the compounds FLP-2 and NTZ (18.3- and 17.2-fold change, respectively) compared to the control group. This increase could be related to a greater energy requirement to survive or proliferate in the presence of compounds that affect metabolism [41,42]. It could also be a response to cell death of the parasite since various studies have related the GAPDH enzyme with apoptosis [41,43].

The *PK* and *PPDK* genes encode enzymes that catalyze the conversion of phosphoenolpyruvate (PEP) to pyruvate with the production of an ATP molecule contributing to the ATP pool that *G. lamblia* needs for its survival [44,45]. The *PPDK* enzyme uses AMP and pyrophosphate (PPi) as substrates to produce ATP. In this manner, the *PPDK* enzyme is essential for the survival of the parasite under anaerobic conditions since it allows it to obtain energy without depending on ADP, which is an adaptive advantage in its microaerophilic environment [45]. The results of gene expression show an increase in the expression of both *PK* and *PPDK*; however, the compounds CNZ-7, CNZ-8, and FLP-2 showed a significant overexpression with 15.8-, 8.2-, and 21.8-fold changes in *PPDK* when compared with the control group.

Hydrolysis of the anhydrous pyrophosphate bond releases high energy (ΔG = 20–30 kJ mol^−1^); this energy converts PPi into a plausible alternative energy currency under conditions where low concentrations of ATP are produced [46,47]. Therefore, PPi and other inorganic phosphates have been suggested as the first energy currencies in the origin of life [48,49]. Probably, the ATP levels in *G. lamblia* are strongly affected by the compounds due to the decreased expression of the *TPI* and *PFK* genes, limiting ATP synthesis; then, PPi could be functioning as an alternative energy currency and as a substrate of the enzyme *PPDK*, consequently increasing the *PPDK* gene expression levels. For example, in plants, it has been observed that, under conditions where the Mg^2+^ concentration increases, particularly under decreased oxygen stress, this leads to a decrease in ATP and an increase in ADP and AMP; then, PPi functions as an energy currency, ensuring the continuous functioning of glycolysis, keeping the metabolism energetically efficient and less dependent on ATP [50]. In addition, it has been mentioned that pyruvate can be used by the parasite as a non-enzymatic antioxidant, which can play a role in response and protection against reactive oxygen species [51].

Regarding the expression profiles determined for the *PFOR* gene, it was found that trophozoites treated with the compounds CNZ-7, FLP-2, and NTZ showed a significant overexpression with 7.4-, 16-, and 14-fold changes with respect to the control group. The *PFOR* gene encodes a protein relevant to the metabolism of *Giardia lamblia* since, through the oxidative decarboxylation of pyruvate to acetyl-CoA and CO_2_, the two electrons generated from the *PFOR* reaction are transferred to ferredoxin, whose enzyme helps in the detoxification processes and the energy production processes of the parasite [52,53]. Previously, it was reported that the PFOR enzyme could be a target of NTZ since NTZ behaves as a catalytic inhibitor of GlG6PD::6PGL [14]. Similarly, CNZ-7 and FLP-2 behave as catalytic inhibitors of G6PD [20]; thus, the increase in the expression of the *PFOR* gene is probably related to the inhibition of the PFOR enzyme, and the parasite attempts to compensate for this inhibition with the higher production of PFOR mRNA [54].

**Figure 5:**
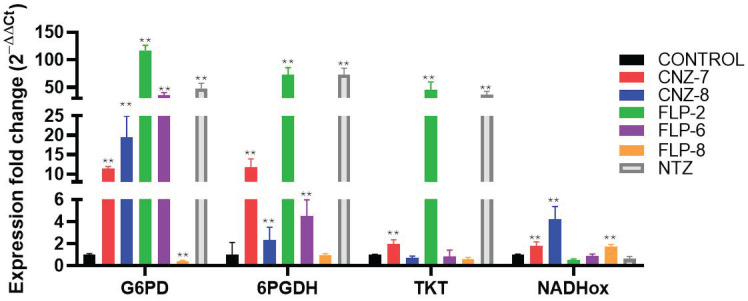
Relative expression of pentose phosphate pathway genes in *Giardia lamblia* determined using RT-qPCR. Gene expression was compared between parasites cultured without treatment (negative control) and trophozoites exposed to compounds, using Aldolase as a reference gene. Each color corresponds to the evaluation of different compounds: red—CNZ-7; blue—CNZ-8; green—FLP-2; purple—FLP-6; orange—FLP-8; gray—NTZ; and black—negative control. Double asterisk (**) indicates a significant difference (*p* < 0.001) in expression. Values represent the mean ± SD of three replicates.

Furthermore, the expression of genes involved in the pentose phosphate pathway was also evaluated, a pathway closely related to various metabolic processes such as nucleotide synthesis, the production of NADPH molecules, which are useful in the response to oxidative stress and lipid synthesis, and the synthesis of intermediates of other pathways such as glyceraldehyde-3-phosphate (G3P) and fructose-6-phosphate [22,55]. The expression of glucose-6-phosphate dehydrogenase (*G6PD*), 6-Phosphogluconolactonase (*6PGL*), 6-phosphogluconate dehydrogenase (*6PGDH*), and transketolase (*TKT*) genes showed a differential expression profile in the presence of the five compounds compared to the control group (Figure 5).

Trophozoites exposed to the compounds CNZ-7, CNZ-8, FLP-2, FLP-6, and NTZ showed a significant increase in the expression of the *G6PD::6PGL* gene with values of 11.4-, 19.4-, 117.4-, 35-, and 48.3-fold change with respect to the control. Notably, it was previously reported that the G6PD enzyme could be a target of NTZ since NTZ behaves as a catalytic inhibitor of *Gl*G6PD::6PGL [14]; likewise, CNZ-7 and FLP-2 behave as the catalytic inhibitors of *Gl*G6PD::6PGL [28]; thus, the increase in the expression of the *G6PD::6PGL* gene is probably related to the inhibition of the *Gl*G6PD::6PGL enzyme, and the parasite attempts to compensate for this inhibition with a higher production of *G6PD::6PGL* mRNA, since G6PD plays a fundamental role in the pentose phosphate pathway (PPP) regulation. Moreover, the genes *6PGDH* and *TKT* showed differential overexpression (*p* < 0.001) when trophozoites were treated with the compound FLP-2 and the NTZ drug.

Nitrothiazole aromatic drugs, such as NTZ, have been reported to induce a significant increase in superoxide radical production; however, they do not induce a change in the activity of the nicotinamide adenine dinucleotide oxidase enzyme (NADHox) [56,57], which probably leads to oxidative imbalance in *G. lamblia*, since one of the enzymes involved in the detoxification of reactive oxygen species in *G. lamblia* is NADHox [55,58,59]. In this work, we determined the expression profiles of the *NAHDox* gene in trophozoites treated with the five compounds and NTZ; the profiles showed different effects on gene expression in the presence of the compounds CNZ-7, CNZ-8, and FLP-8, which increased gene expression by 1.7, 4.2, and 1.7 times more than the control, while for FLP-2 and NTZ, the expression was lower. Although *G. lamblia* does not have classic oxidative stress response systems such as catalase, superoxide dismutase, or glutathione reductase, it does have the enzyme thioredoxin reductase, which is another enzyme that helps to respond to oxidative stress in the cell [60]. The difference in *NADHox* expression in the presence of the compounds could be because *G. lamblia* does not depend exclusively on the NADH oxidase enzyme to respond to oxidative stress. It has been proposed that the thioredoxin system (thioredoxin reductase (TrxR)/thioredoxin (Trx)), as well as peroxiredoxins (Prx1), constitutes a pathway widely used by the parasite as a defense against oxidizing conditions [61,62]. Although in the present work, we did not analyze whether these antioxidant mechanisms intervene in protecting trophozoites exposed to compounds from oxidative damage, it has been proposed that TrxR is an important target of metronidazole (nitro-drug) [63]. *G. lamblia* could probably make use of these mechanisms; however, it would be important to evaluate them. The last gene analyzed in the PPP was *TKT*, an enzyme that participates in the non-oxidative phase of the pathway; CNZ-8, FLP-6, and FLP-8 decreased their expression by 1.4, 1.2, and 1.7 times compared to the control; on the other hand, CNZ-7, FLP-2, and NTZ increased the expression by 1.9, 45.1, and 36.1 times compared to the control, suggesting that trophozoites may require a higher availability of glycolytic intermediates. This is because one of the products of the non-oxidative phase of the pentose phosphate pathway serves as an intermediate in this metabolic route.

**Figure 6:**
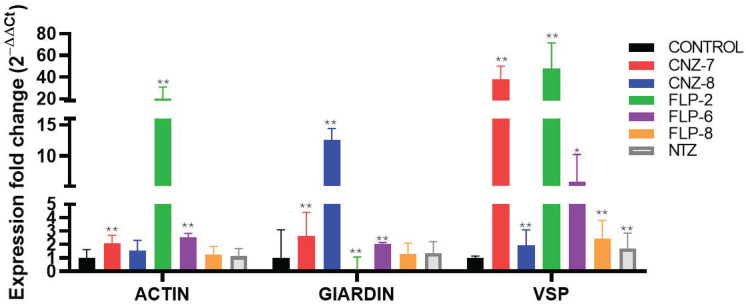
Relative expression of structural genes in *Giardia lamblia* determined using RT-qPCR. Gene expression was compared between parasites cultured without treatment (negative control) and trophozoites exposed to compounds CNZ-7, CNZ-8, FLP-2, FLP-6, FLP-8, and nitazoxanide, using Aldolase as a reference gene. Each color corresponds to the evaluation of different compounds: red—CNZ-7; blue—CNZ-8; green—FLP-2; purple—FLP-6; orange—FLP-8; gray—NTZ; and black—negative control. The asterisk (*) indicates a significant difference (*p* < 0.05) in expression, and double asterisk (**) indicates a significant difference (*p* < 0.001) in expression. Values represent the mean ± SD of three replicates.

Finally, the expression of genes encoding proteins relevant to the cytoskeletal structure and stability of the parasite membrane were analyzed; see Figure 6. The expression of actin and δ-giardin, the cytoskeletal proteins essential for the structure, mobility, and cell division of the parasite, was evaluated. Actin plays a relevant role in the ventral disc since it contains actin filaments that help in its assembly and stability, allowing Giardia to resist intestinal flow and remain anchored to its infection site, which in turn is associated with the adhesion of the parasite. As for giardin, it is a protein that is only found in *G. lamblia*, and its function is to give rigidity and structural stability to the cells; it is closely associated with the ventral disc and the flagella, which is why it is relevant for the motility of the parasite [64]. The expression of *ACT* increased in the CNZ-7 and FLP-6 treatments and had 2.1 and 2.5 times higher expression compared to the control; contrastingly, FLP-2 expression decreased by 10 times. *GIA* expression increased in the presence of CNZ-7 by 2.6 times, CNZ-8 by 12.5 times, FLP-2 by 20.1 times, and FLP-6 by 2.6 times compared to the control; FLP-8 and NTZ did not show changes in expression. Finally, the expression of VSP increased by 47.8, 5.9, and 2.4 times for compounds FLP-2, FLP-6, and FLP-8 with respect to the control, which indicates that these compounds probably affect structural stability to a greater extent.

In general, the expression analysis performed showed significant changes in the expression levels of several metabolic genes in response to the exposure of *G. lamblia* to the IC_50_ concentration of each compound for 48 h. The genes analyzed showed variations in their transcription, suggesting a possible relationship between the regulation of these genes and cell death in the parasite. These results support the hypothesis that the alteration in the expression of metabolic genes may contribute to the efficacy of the compounds in eliminating *G. lamblia*. It was observed that the compounds belonging to the FLP family, FLP-2, FLP-6, and FLP-8, which were the most potent and showed the lowest IC_50_ values, cause greater alterations in genes of the enzymes of the PPP pathway, which in turn cause greater alterations in metabolism and affect cell homeostasis, thus altering the expression of other proteins relevant to the survival of the parasite such as structural proteins.

### 2.4. Cell Damage Caused by the Compound FLP-2 in Giardia lamblia Trophozoites

**Figure 7:**
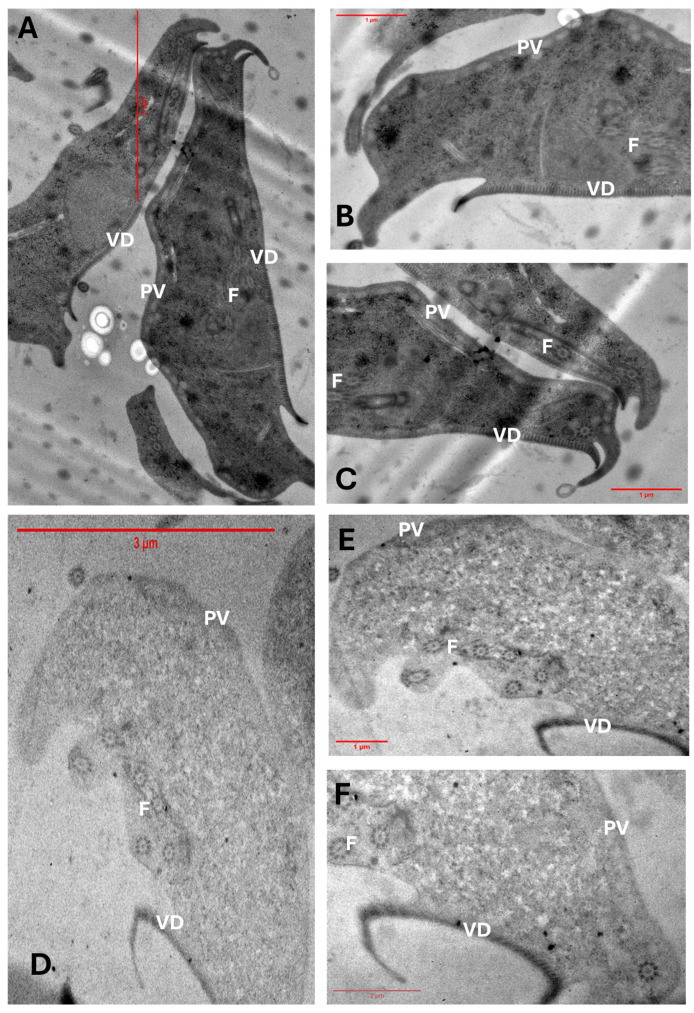
Transmission electron microscopy (TEM) images of *G. lamblia* trophozoites. (**A**–**C**) Untreated trophozoites displaying well-preserved cellular structures, including ventral disc (VD), flagellar axonemes (F), and peripheral vesicles (PV). (**D**–**F**) Trophozoites exposed to FLP-2 at its IC_50_ concentration for 24 h, showing extensive ultrastructural damage, including ventral disc deformation, cytoplasmic translucency, altered flagellar axonemes, and nuclear disorganization.

To assess the ultrastructural effects of FLP-2 on *G. lamblia* trophozoites, we performed transmission electron microscopy (TEM) analysis on untreated and treated cells. In the control group (Figure 7A–C), trophozoites exhibited characteristic cellular structures, including well-defined nuclei (N), ventral disc (VD), flagellar axonemes (F), and peripheral vesicles (PV). The cytoplasm appeared electron-dense with intact membranes, and the ventral disc maintained its normal architecture.

In contrast, trophozoites treated with FLP-2 at its IC_50_ concentration for 24 h (Figure 7D–F) displayed significant morphological alterations. The cytoplasmic content became less electron-dense, resulting in a translucent appearance. Notable structural disorganization was observed, including the deformation and partial detachment of the ventral disc, which is crucial for adhesion. The flagellar axonemes exhibited alterations, and peripheral vesicles appeared less defined. Additionally, nuclear morphology was affected, suggesting potential damage to intracellular integrity. These findings indicate that FLP-2 disrupts key cellular structures in *G. lamblia*, impairing its viability and potentially affecting its ability to colonize the host.

Thus, the deformation and detachment of the VD strongly suggest structural damage to the cytoskeleton, specifically to microtubules and associated proteins. Additionally, such structural damage suggests that FLP-2 may target cytoskeletal components, impairing the parasite’s ability to attach to host surfaces, which is essential for its survival in the intestine.

### 2.5. Elucidation of the Effect of Compounds on Drug-Resistant Strains

**Figure 8:**
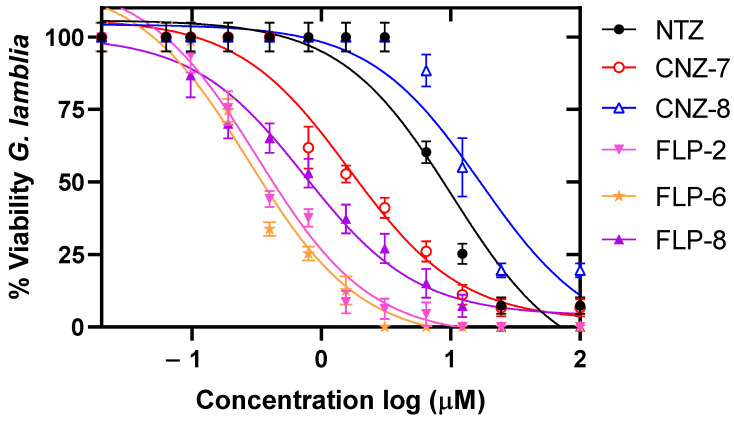
Growth curves of *G. lamblia* culture strain resistant to NTZ in the presence of compounds CNZ-7, CNZ-8, FLP-2, FLP-6, and FLP-8, and the drug NTZ. Trophozoites were incubated with the compounds for 48 h at 37 °C using increasing concentrations of each compound. The IC_50_ concentration (μM) of each compound was subsequently determined using the IC_50_ Calculator software (https://www.aatbio.com/tools/ic50-calculator).

Finally, we evaluated the effect of the five compounds on the viability of *G. lamblia* strain resistant to nitazoxanide. Trophozoites were incubated with different concentrations of the compounds for 48 h, and the percentage of viability was determined for each condition. As shown in Figure 8, a concentration-dependent behavior was observed. The results indicated that CNZ-8, FLP-6, and FLP-8 showed higher IC_50_ values (13.7, 0.54, and 1.19 µM, respectively) for the nitazoxanide-resistant strain compared to the ATCC strain. However, although the IC_50_ values of FLP-6 and FLP-8 were higher, these values are below those determined for NTZ (IC_50_ value = 7.29 µM), indicating 6 to 13 times more potency than this drug (Table 2).

In contrast, the compounds CNZ-7 (IC_50_ value = 2.5 µM) and FLP-2 (IC_50_ = 0.37 µM) showed a decrease in IC_50_, reducing the IC_50_ for the nitazoxanide-sensitive strain by 2 and 5 times, respectively. This effect could be attributed to the specific changes in the resistant strain that make it more susceptible to these compounds. The findings suggest that the compounds evaluated in this study inhibit the viability of nitazoxanide-resistant strain cultures, reinforcing their potential as promising antigiardial agents.

## 3. Materials and Methods

### 3.1. Synthesis of Compounds FLP-2, FLP-6, and FLP-8

In a 50 mL round-bottom flask equipped with magnetic stirring and cooled in an ice bath, 1 equivalent of 2-amino-5-nitrothiazole was dissolved in 5 mL of anhydrous CH_2_Cl_2_. Subsequently, 1.1 equivalents of Et3N were added, and the reaction was stirred for 5 min. Following this, 1.1 equivalents of the appropriately substituted acyl chloride were added dropwise using a pressure-equalized addition funnel. Upon the completion of the addition, the ice bath was removed, and the addition funnel was replaced with a Vigreux column. The reaction progress was monitored via TLC until the full conversion of the starting material. The reaction mixture was then concentrated using a rotary evaporator at 45 °C under reduced pressure. The resulting solid was washed with water and filtered under suction, yielding the compounds FLP-2, FLP-6, and FLP-8, which were subsequently purified by recrystallization using a solvent specific to each compound. The chemical structures of the synthesized compounds were confirmed based on their spectral data: nuclear magnetic resonance of proton (^1^H NMR) and Carbon-13 (^13^C NMR), as well as mass spectra obtained with fast-atom bombardment mass spectrometry (MS FAB^+^).

*N*-(5-Nitro-1,3-thiazol-2-yl)pentanamide (FLP-2). Yield: 67%, recrystallized from ethanol. Mp: 155 °C [65]. ^1^H NMR (200 MHz, CDCl_3_) d: 0.72–0.84 (3H, m, CH_3_), 1.17–1.27 (2H, m, CH_2_), 1.58–1.67 (2H, m, CH_2_), 2.42–2.46 (2H, m, CH_2_), 8.19 (1H, s, H-4), 12.33 (1H, s, NH) ppm. ^13^C NMR (50 MHz, CDCl_3_) δ: 13.9 (CH_3_), 22.4 (CH_2_), 27.1 (CH_2_), 35.6 (CH_2_), 141.7 (C-4), 142.7 (C-5), 162.4 (C-2), 173.2 (CO) ppm. MS (FAB^+^): *m*/*z* 230 (M+H^+^).

3-Methoxy-*N*-(5-nitro-1,3-thiazol-2-yl)benzamide (FLP-6). Yield: 92.5%, recrystallized from methanol. Mp: 218.9–221.1 °C. ^1^H NMR (200 MHz, CDCl_3_) δ: 3.79 (s, 3H, OCH_3_), 7.05 (dd, *J* = 2.6, *J* = 2.2 Hz, 1H, H-4′), 7.32 (t, *J* = 8.8 Hz, 1H, H-5′), 7.6 (s, 1H, H-2′), 7.64 (d, *J* = 8, 1H, H-6′), 8.30 (s, 1H, H-4) ppm. ^13^C NMR (50 MHz, CDCl_3_) δ: 55.1 (OCH_3_), 127.0 (C-5), 112.6 (C-2′), 119.4 (C-4′), 120.4 (C-6′), 125.7 (C-5′), 129.2 (C-5), 131.7 (C-1′), 140.9 (C-4), 142.4 (C-3′), 159.2 (C-2), 162 (C=O) ppm. MS (FAB^+^): *m*/*z* 280 (M+H)^+^.

3-Nitro-*N*-(5-nitro-1,3-thiazol-2-yl)benzamide (FLP-8). Yield: 17.9%, recrystallized from ethanol. Mp: 142.1–143.1 °C. ^1^H NMR (200 MHz, CDCl_3_) δ: 7.59 (t, *J* = 8.1 Hz, 1H, H-5′), 8.2 (m, 1H, H-6′), 8.39 (s, 1H, H-4), 8.52 (d, *J* = 7.6 Hz, 1H, H-4′), 9.02 (m, 1H, H-2′) ppm. ^13^C NMR (50 MHz, CDCl_3_) δ: 126.2 (C-2′), 128.6 (C-4′), 130.7 (C-5′), 134.4 (C-6′), 139.7 (C-1′), 142.7 (C-5), 144.3 (C-3′), 148.8 (C-4), 152.9 (C-2), 175.9 (C=O) ppm. MS (FAB^+^): *m*/*z* 295 (M+H)^+^.

### 3.2. Determination of the IC_50_ Value of Inhibitors of Giardia lamblia Culture

The compounds CNZ-7 and CNZ-8, from an in-house library collection, and the synthesized compounds FLP-2, FLP-6, and FLP-8 inhibited the growth of the *G. lamblia* parasite culture by more than 98%; thus, their IC_50_ value (μM) was determined to perform subsequent tests. The IC_50_ is defined as the concentration of compound at which 50% of the viability of *G. lamblia* cultures is inhibited.

Viability analysis was performed on *G. lamblia* strain ATCC 30957 grown in TYI-S-33 medium supplemented with 10% fetal bovine serum (FBS) and an antibiotic. Pre-culture was performed in 16 × 150 mm borosilicate tubes with a medium inoculated with 2 × 10^5^ trophozoites and incubated at 37 °C for 48 h. For the recovery of the trophozoites, the vial was incubated in ice for 8 min and centrifuged for 6 min at 2000 rpm. The concentrated trophozoites were suspended in 1 mL of phosphate-buffered saline (PBS), and the number of trophozoites was counted by placing a 1:1 volume ratio of cell culture and trypan blue dye (4 g/L); then, a homogeneous drop of the mixture was taken and placed in the Neubauer chamber, and the total cells were counted.

The curves were plotted for concentrations of 0–50 µM of each compound. The assay was performed by placing 1.5 × 10^5^ cells in 1.5 mL microcentrifuge tubes with a supplemented medium and different concentrations of each compound (0–50 µM). The negative control was performed under the same conditions without a compound, and the drugs NTZ and MTZ were used as positive controls. The samples were incubated for 48 h at 37 °C. At the end of the incubation, the cell viability count was performed using the trypan blue technique. The viability calculation was performed following the formula nv/(nv + nm), where nv represents the live cells and nm represents the dead cells; the viability value obtained was expressed as a percentage. The IC_50_ value of each compound was determined using the IC_50_ Calculator software (https://www.aatbio.com/tools/ic50-calculator).

### 3.3. Cytotoxicity Assessment of Compounds in Caco-2 and HT-29 Cell Cultures

The cytotoxicity assay was performed to evaluate the effect of the selected compounds on human cells. Colon cells were used because they are cell lines like those present in the microenvironment where *G. lamblia* colonizes, which is the small intestine of the host. The assay was performed using the human cell lines Caco-2 (ATCC HTB-37) and HT-29 (ATCC HHTB-38). Cell viability was determined using the Cell Proliferation Kit II (XTT, St. Louis, MO, EE. UU.). A cell suspension of 2 × 10^3^ cells/mL was prepared in Dulbecco’s modified Eagle’s medium (DMEM) supplemented with 10% inactivated fetal bovine serum and an antibiotic (1% penicillin/streptomycin, 5000 U/mL, in a volume of 100 µL). The suspension was added to a 96-well plate. The plates were incubated for 24 h at 37 °C in a 5% CO_2_ atmosphere. The medium in each well was replaced with 100 µL of medium-containing compounds (CNZ-7, CNZ-8, FLP-2, FLP-6, and FLP-8) at concentrations of 250, 125, 62, 32, 15, 7, and 3 µM. The cells were incubated for 48 h at 37 °C in a 5% CO_2_ atmosphere. Cell viability was determined using the following steps. The medium in each well was replaced with 100 µL of fresh medium, and 50 µL of XTT labeling mix was added per well. The reaction was incubated for four hours at 37 °C and 5% CO_2_. After incubation, the absorbance of the samples on the plate was measured at 450 nm. Each compound was assayed in triplicate in three independent experiments. CC_50_ values (μM) were determined using the nonlinear regression function of GraphPad Prism version 8 for Windows.

### 3.4. Evaluation of the Effect of Compounds on the Expression Levels of G. lamblia Genes Using Quantitative RT-qPCR

To determine the alterations in the parasite’s metabolism and cellular processes induced by the compounds, the expression levels of various genes encoding metabolic, structural, and oxidative stress response proteins were evaluated. *G. lamblia* strain ATCC 30957 was grown by following previously established culture parameters. The sequences for primer design were obtained from GenBank, and the accession number for each gene was provided in Table 3. The genes that were evaluated are listed in Table 3.

Treatments were performed with 1 × 10^5^ trophozoites and incubated for 48 h at 37 °C in the presence of the IC_50_ of each compound. The culture tube was incubated on ice for 15 min and then centrifuged at 2000 rpm for 6 min to concentrate the cell pellet. Cell counting was performed using the trypan blue method. A 500 µL volume of Trizol ^®^ was added, and it was gently homogenized; then, 60 µL of chloroform was added, and it was homogenized for 10 s with a vortex and incubated on ice for 3 min. Afterward, it was centrifuged at 12,000 rpm for 10 min, the aqueous phase was transferred to a new tube, 200 µL of isopropanol was added, and it was incubated for 3 h on ice. The sample was then centrifuged for 10 min at 12,000 rpm, and the supernatant was discarded. A 600 µL volume of ethanol (70%) was added, homogenized, and centrifuged. In the end, the supernatant was removed, and the sample was centrifuged again to dry the excess alcohol. The pellet was dried. Finally, it was resuspended in 25 µL of sterile MilliQ water. The concentration and purity of the extracted RNA were determined using the NanoDrop^®^ Spectrophotometer ND-1000 (NanoDrop Technologies, Wilmington, DE, USA). The extracted RNAs exhibited a 260/280 nm absorbance ratio ranging from 2.0 to 2.2. RNA purity and integrity were confirmed using 0.8% (*w*/*v*) agarose gels under denaturing conditions.

The synthesis of cDNA was carried out with 1 mg of purified RNA, and 1 U of DNase II was added (Thermo Thermo Fisher Scientific, Waltham, MA, USA). First-strand cDNA synthesis was carried out using 1 μg of DNase-treated RNA, combined with a 10 mM dNTP mix, oligo(dT)18 primer, and RevertAid reverse transcriptase (Thermo Scientific) in a final reaction volume of 20 μL. The reaction mixture was incubated at 42 °C for 60 min, followed by heat inactivation at 70 °C for 10 min. The synthesized cDNA was stored at −20 °C until further use. The level of purity and concentration of the cDNA were determined using the NanoDrop^®^ Spectrophotometer ND-1000 (NanoDrop Technologies, Wilmington, DE, USA).

Specific oligonucleotides were designed to determine the expression levels of the gene’s phosphofructokinase, pyruvate kinase, glucose-6-phosphate dehydrogenase::6-phosphogluconolactonase, 6-phosphogluconate dehydrogenase, and transketolase. The design was based on gene sequences available in the National Center for Biotechnology Information (NCBI) database, adhering to the following criteria: an oligonucleotide length of 18–20 bp, a melting temperature (Tm) of 61 °C, a GC content of approximately 40%, and an amplicon size between 80 and 200 bp (Table 4). The oligonucleotides were synthesized by the Sequencing and Synthesis Unit of the Institute of Biotechnology, UNAM.

Additionally, previously reported primers that were used for the gene expression analysis of *G. lamblia* were also obtained (Table 4). These primers target genes encoding surface structural protein, giardin, actin, pyruvate:ferredoxin oxidoreductase, pyruvate phosphate dikinase, NADH oxidase, triose phosphate isomerase, glyceraldehyde-3-phosphate dehydrogenase, and Aldolase.

Amplification was performed using a StepOne™ Real-Time PCR System and the Fast SYBR^®^ Green Master Mix kit (Applied Biosystems, Foster City, CA, USA) under the following conditions: an initial denaturation at 95 °C for 30 s, followed by 40 cycles of 95 °C for 3 s and 60 °C for 30 s. The melt curve analysis was conducted post-amplification by gradually increasing the temperature from 60 °C to 95 °C. To determine the expression levels, 100 ng of cDNA from the control trophozoites and from each of the cultures subjected to treatments with the compounds CNZ-7, CNZ-8, FLP-2, FLP-6, FLP-8, and NTZ were used. Normalization was performed using the geometric mean of cycle threshold (Ct) values, and the relative expression levels of the target genes were calculated using the 2^−ΔΔCt^ method [67]. Each reaction was performed in five replicates for all analyzed genes and strains. Results are presented as mean values ± standard deviation (SD).

### 3.5. Evaluation of Pharmacokinetic and Physicochemical Parameters of Selected Antigiardial Compounds

To evaluate the molecules as potential therapeutic agents, their obeyance of Lipinski physicochemical rules and their ADMET pharmacokinetic parameters were determined, which were calculated using computer servers in which the SMILES notation of the selected compounds was used. The physicochemical parameters were calculated using the Chemicalize online server (https://chemicalize.com/app/calculation) (accessed on 24 November 2024), and the ADMET parameters were evaluated in the free software ADMETlab 3.0 (https://admetlab3.scbdd.com/server/screening), (accessed on 24 November 2024).

### 3.6. Evaluation of Cell Damage Using Transmission Electron Microscopy

To determine the cellular damage caused by the most effective compound (FLP-2) in the parasite *G. lamblia*, its effect on trophozoites was analyzed using transmission electron microscopy. Tubes of 1.5 mL containing TYI-S-33 medium were prepared, in which 2 × 10^6^ trophozoites were placed and exposed to the IC_50_ concentration of FLP-8, along with a negative control. The trophozoites were incubated for 24 h at 37 °C and then collected by cooling them in an ice bath for 10 min and centrifuging at 2000 rpm for 7 min. The samples were sent for preparation and observation at the Instituto de Fisiología Celular, UNAM. The analysis was performed using a JEOL-JEM-1200 transmission electron microscope (JEOL LTD, Tokyo, Japan) equipped with a GATAN CCD camera at the Imaging Unit of the Institute of Cellular Physiology, UNAM.

### 3.7. Giardicidal Activity of Selected Compounds in Nitazoxanide-Resistant Strain

To evaluate the effect of the compounds CNZ-7, CNZ-8, FLP-2, FLP-6, and FLP-8 on a drug-resistant *G. lamblia* strain, an assay was conducted to determine their IC_50_ values in a strain isolated from a patient who exhibited resistance to NTZ, as reported by Reyes- Vivas et al. [68]. A pre-culture at confluence was used as a starting point, and a concentration–response curve was plotted using an initial compound concentration of 25 μM in a final volume of 1.5 mL of TYI-S-33 medium, followed by serial dilutions of the compound. Each treatment was inoculated with 1.5 × 10^5^ trophozoites and incubated for 48 h at 37 °C. At the end of the incubation, the number of trophozoites was determined using the trypan blue assay and by counting in a Neubauer chamber. The viability percentage at each compound concentration was calculated using the negative control as a reference.

## 4. Conclusions

In this study, nitazoxanide analog compounds demonstrated effectiveness against *Giardia lamblia* trophozoites. The highest antigiardial activity was observed for FLP-2, FLP-6, and FLP-8, as their IC_50_ values were lower than those of the currently used drugs. Additionally, these compounds were effective against drug-resistant strains. Given that giardiasis remains a significant public health concern, FLP-2, FLP-6, and FLP-8 represent promising antigiardial drug candidates. Further studies are required to evaluate their safety and efficacy, which will be crucial for their potential development as alternative giardiasis treatments.

## Figures and Tables

**Table 1:** Pharmacokinetic predictive values were calculated with ADMETLab 3.0 for compounds CNZ-7, CNZ-8, FLP-2, FLP-6, FLP-8, and nitazoxanide. Prediction probability values are shown with six symbols: 0–0.1 (---), 0.1–0.3 (--), 0.3–0.5 (-), 0.5–0.7 (+), 0.7–0.9 (++), and 0.9–1.0 (+++).

Model		Compounds					
		CNZ-7	CNZ-8	FLP-2	FLP-6	FLP-8	NTZ
A	Gastrointestinal absorption	(---)	(---)	(---)	(---)	(---)	(---)
	Caco-2 permeability	−4.839	−4.686	−4.798	−4.789	−4.908	−5.038
D	Plasma protein binding	98%	93.5%	96.4%	97.8%	96.6%	97.2%
	Blood–brain barrier permeability	(---)	(---)	(++)	(---)	(---)	(+++)
	Volume of distribution	1.899 L/kg	0.526 L/kg	1.32 L/kg	1.085 L/kg	0.633 L/kg	0.478 L/kg
M	CYP3A4 substrate	(---)	(-)	(---)	(---)	(---)	(---)
	CYP2D6 substrate	(+++)	(+++)	(+++)	(+)	(-)	(---)
E	CL plasma	3.832 mL/min/Kg	1.697 mL/min/Kg	4.826 mL/min/Kg	4.381 mL/min/kg	2.615 mL/min/kg	2.529 mL/min/kg
	Half-life (T_½_)	<3 h	<3 h	<3 h	<3 h	<3 h	<3 h
T	Blockers hERG	(--)	(---)	(--)	(--)	(---)	(--)
	Rat oral acute toxicity	(+++)	(+++)	(+++)	(+++)	(+++)	(+++)
	Carcinogenesis	(+++)	(+++)	(+++)	(+++)	(+++)	(++)

**Table 3:** Genes analyzed in this study.

Gene Symbol	Gene Name	Length (bp)	Function	Accession Number
*PFK*	Phosphofructokinase	1635	Transferase in glycolysis	XM_001707455.1
*TPI*	Triose phosphate isomerase	764	Oxidoreductase in glycolysis	XM_001706778
*GAPDH*	Glyceraldehyde-3-phosphate dehydrogenase	1224	Oxidoreductase in glycolysis	XM_001703983
*PK*	Pyruvate kinase	1662	Oxidoreductase in glycolysis	XM_001709477.1
*PPDK*	Pyruvate phosphate dikinase	2655	Oxidoreductase in glycolysis	XM_001705520.1
*ALDO*	Aldolase	972	Oxidoreductase in glycolysis	XM_001709998
*G6PD*	Glucose-6-phosphate dehydrogenase	2229	Oxidoreductase in PPP	XM_001704389.1
*6PGDH*	6-Phosphogluconate dehydrogenase	1416	Oxidoreductase in PPP	XM_001704391.1
*TKT*	Transketolase	2160	Transferase in PPP	XM_001704562.1
*NADHox*	NADH oxidase	1377	O2 detoxifying enzyme	XM_001707922
*PFOR*	Pyruvate:ferredoxin oxidoreductase	3762	Oxidoreductase	XM_001708652.1
*GIA*	Giardin	1091	Cytoskeletal structural protein	AF331827
*ACT*	Actin	1128	Cytoskeletal structural protein	AF331826
*VSP*	Variant surface protein	702	Membrane protein	U89152

**Table 4:** Primers designed for evaluating expression levels using RT-qPCR.

Gene	5′-3′ Sequence	Length (bp)	Function
*δ-GIA*, giardin [66]	Fw 5′ AGGACGACCAGGAGGAGAA-3′ Rv 5′ ACGGGTAAAGGCACAATTCA-3′	74	Structural
*ACT*, actin [66]	Fw 5′ TTGCCGTACCTGCCTTCTAT Rv 5′ GCCCGGAACTGTAGAGAGC	60	Structural
*VSP*, variant-specific surface [66]	Fw 5′ GCGAAAGTGATAGCAATGGG Rv 5′ TGAGGTAACAGAGGACGGAGC	60	Structural
*P-FOR*, pyruvate oxidoreductase [54]	Fw 5′ CTACGACATTGACTTTGCTG-3′ Rv 5′ CCCATCTTCTTGTCCTTGAC-3′	180	Energy production
*NADH*, oxidase [66]	Fw 5′ GCACCATATGGCTTCAACGG Rv 5′ CAGGCCTGTCCGTGTCATTA	98	Oxidative stress
*ALD*, Aldolase [66]	Fw 5′ GAGTCCGTGAAGATGGCGA Rv 5′ GTCCCAAGTTCAGCCTCCAC	149	Glycolysis
*TPI*, triose phosphate isomerase [66]	Fw 5′ AGGAGCTCGGAGAGTCCAA Rv 5′ ACACGGGCTCGTAAGCAAT	60	Glycolysis
*GAPDH*, glyceraldehyde-3-phosphate [66]	Fw 5′ CATGGAGCGTGCCTACTT Rv 5′ CACTCCAAGACCACATCC	237	Glycolysis
*PPDK*, pyruvate phosphate dikinase [66]	Fw 5′ TTGGAAACACAGGCGATGAC Rv 5′ TCATCATAGCACGCCTTCCA	196	Glycolysis
*G6PD*, glucose-6-phosphate dehydrogenase	Fw 5′- CTACCTTCACAAGGACAC-3′ Rv 5′- ATACCGTCCTTAATACGA -3′	87	PPP
*6PDH*, 6-phosphogluconate dehydrogenase	Fw 5′ CTCGACATGATCCAGACTG-3′ Rv 5′ TCATAGGTGTGAGCTCCAA-3′	80	PPP
*TKT*, transketolase	Fw 5′ AAGATCACCATACACGGC-3′ Rv 5′ ACGGGATAGGCATACGATA-3′	96	PPP
*PFK*, phosphofructokinase	Fw 5′ ATCTCTCAGATTGAAACG-3′ Rv 5′ AGTGATAGAGCGGAGTAA-3′	97	Glycolysis
*PK*, pyruvate kinase	Fw 5′ AGGTGTGGATAAGAATCA-3′ Rv 5″ GATCATTCCTGCTATGAC-3′	97	Glycolysis

## Data Availability

Data are contained within the article.
